# Current State and Future Directions in the Diagnosis of Amyotrophic Lateral Sclerosis

**DOI:** 10.3390/cells12050736

**Published:** 2023-02-24

**Authors:** Maximilian Vidovic, Lars Hendrik Müschen, Svenja Brakemeier, Gerrit Machetanz, Marcel Naumann, Sergio Castro-Gomez

**Affiliations:** 1 Department of Neurology, University Hospital Carl Gustav Carus, Technische Universität Dresden, 01307 Dresden, Germany; 2 Department of Neurology, Hannover Medical School, 30625 Hannover, Germany; 3 Department of Neurology and Center for Translational Neuro and Behavioral Sciences (C-TNBS), University Hospital Essen, 45147 Essen, Germany; 4 Department of Neurology, Klinikum Rechts der Isar, Technical University of Munich, 81675 Munich, Germany; 5 Translational Neurodegeneration Section “Albrecht Kossel”, Department of Neurology, University Medical Center, University of Rostock, 18147 Rostock, Germany; 6 Department of Neurodegenerative Disease and Geriatric Psychiatry/Neurology, University Hospital Bonn, 53127 Bonn, Germany; 7 Institute of Physiology II, University Hospital Bonn, 53115 Bonn, Germany; 8 Department of Neuroimmunology, Institute of Innate Immunity, University Hospital Bonn, 53127 Bonn, Germany

**Keywords:** amyotrophic lateral sclerosis, ALS, motor neuron disease, MND, diagnosis, diagnostics

## Abstract

Amyotrophic lateral sclerosis (ALS) is a fatal neurodegenerative disease characterized by loss of upper and lower motor neurons, resulting in progressive weakness of all voluntary muscles and eventual respiratory failure. Non-motor symptoms, such as cognitive and behavioral changes, frequently occur over the course of the disease. Considering its poor prognosis with a median survival time of 2 to 4 years and limited causal treatment options, an early diagnosis of ALS plays an essential role. In the past, diagnosis has primarily been determined by clinical findings supported by electrophysiological and laboratory measurements. To increase diagnostic accuracy, reduce diagnostic delay, optimize stratification in clinical trials and provide quantitative monitoring of disease progression and treatment responsivity, research on disease-specific and feasible fluid biomarkers, such as neurofilaments, has been intensely pursued. Advances in imaging techniques have additionally yielded diagnostic benefits. Growing perception and greater availability of genetic testing facilitate early identification of pathogenic ALS-related gene mutations, predictive testing and access to novel therapeutic agents in clinical trials addressing disease-modified therapies before the advent of the first clinical symptoms. Lately, personalized survival prediction models have been proposed to offer a more detailed disclosure of the prognosis for the patient. In this review, the established procedures and future directions in the diagnostics of ALS are summarized to serve as a practical guideline and to improve the diagnostic pathway of this burdensome disease.

## 1. Introduction

The diagnosis of amyotrophic lateral sclerosis (ALS) remains an enormous challenge not only to general physicians, but also to specialized neurologists. ALS, a fatal neurodegenerative and the most frequent motor neuron disease (MND), is primarily characterized by progressive weakness of voluntary muscles due to degenerating motor neurons in the brain, brainstem and spinal cord. Considered to be a multisystem disorder, it can also be accompanied by non-motor symptoms, such as behavioral and cognitive impairment, and even manifest as an overlap syndrome with signs of frontotemporal dementia (FTD), known as ALS-FTD [1,2,3].

The clinical, genetic and neuropathological heterogeneity, and the resemblance to other neuromuscular diseases, especially early in the disease’s course, commonly described as ALS mimics, often requires the application of additional diagnostic methods. Given the short median survival of 2 to 4 years [3,4] and a diagnostic delay of 10 to 16 months [5], there is an urgent need for optimizing diagnostic accuracy to yield a faster and more reliable recognition of the disease. This, in turn, may provide access to novel therapeutic agents and participation in clinical trials at an early disease stage.

In recent decades, the diagnosis of ALS was mainly based on clinical findings with support of electrophysiological, imaging and laboratory techniques to exclude other diseases. Recent efforts have focused on establishing simplified, more practice-oriented diagnostic guidelines, as well as investigating the potential of specific fluid biomarkers, functional brain imaging and additional measures, such as predictive models, to hasten diagnosis, improve diagnostic accuracy and help outline the disease trajectory for the individual patient.

This review aims to present the current state of ALS diagnosis and outline how recent findings and evolving concepts may show where it is headed in the future.

## 2. Clinical Presentation

It is essential to record the clinical findings and their course as accurately as possible. Therefore, a thorough exploration of the patient’s history of symptoms and a complete physical and neurological examination should be considered as the first step in the diagnosis of ALS. 

The clinical hallmarks of ALS are related to the impairment of voluntary muscles, resulting in progressive weakness of the limbs, speech and swallowing dysfunction and respiratory failure with concomitant signs, such as muscle atrophy, fasciculations and increased muscle tone. These motor dysfunctions are derived from the combined impairment of the upper motor neurons (UMN) in the motor cortex of the brain and the lower motor neurons (LMN) in the brainstem and the spinal cord [1,3]. Though generally unaffected, there is also evidence of oculomotor, sphincter and autonomic dysfunction [6,7]. Initially, the disease typically presents itself either with asymmetrical focal muscle weakness in the upper or lower limbs in spinal onset ALS or with speech and swallowing difficulties due to facial, tongue and pharyngeal muscle weakness in bulbar-onset ALS [2]. Disease propagation preferably follows an organized contiguous pattern with first symptoms in one limb, subsequently spreading to the contralateral limb and, later, to adjacent regions [8]. However, disease progression is highly variable and frequently shows a non-linear decline [9]. The anatomically separated body regions (or segments) are defined as the bulbar, cervical, thoracic and lumbar regions, with the latter three comprising the spinal regions [10].

**Figure 1:**
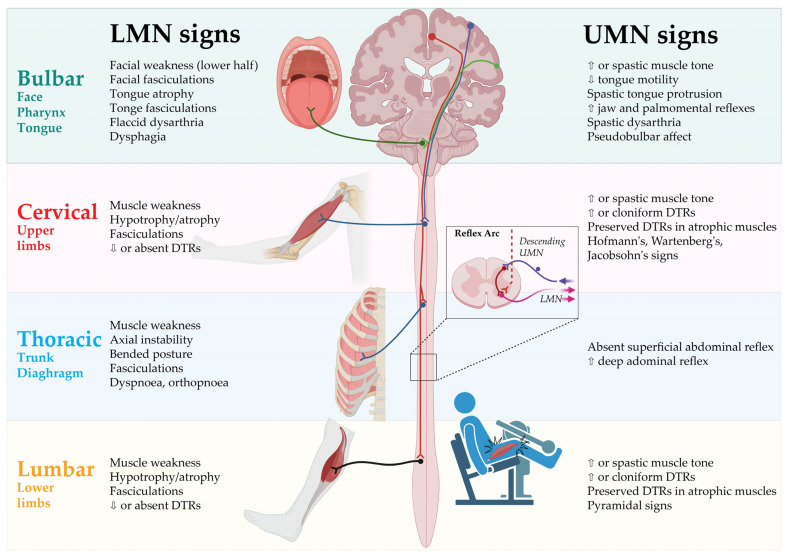
Clinical signs of UMN and LMN involvement according to body regions. DTR: Deep tendon reflex; LMN: Lower motor neurons; UMN: Upper motor neurons. Created by Castro-Gomez with “BioRender.com”; accessed on 4 February 2023.

The clinical signs of UMN and LMN involvement according to each of the body regions are presented in Figure 1.

The notable clinical heterogeneity of motor manifestations frequently leads to controversy in the description of phenotypes as opposed to the determination of the diagnosis of ALS. Distinguishing these phenotypes is of relevance, as they are associated with various disease progression rates and survival times [11].

The different clinical phenotypes of ALS are generally described with regard to the extent of UMN and LMN impairment, its distribution and progression to the different body segments [2,11].

### 2.1. Spinal-Onset ALS

ALS with spinal onset is defined by focal weakness in distal muscle groups of the limbs and simultaneous UMN and LMN involvement. With up to 82% of all ALS patients [12], it is the most common phenotype, termed typical or classical ALS. Distal segments of the upper or lower limbs are affected in a focal manner at the onset of the disease. Characteristically, thenar eminence with the abductor pollicis brevis (APB) and the first dorsal interosseus (FDI) muscles are more affected compared to the hypothenar muscle abductor digiti minimi (ADM), referred to as split-hand syndrome [13]. Onset in the dominant hand is thought to be predominant [14]. Lower-limb weakness typically becomes apparent as it causes an unsteady gait due to weak foot dorsiflexion [15]. Notably, UMN dysfunction is not always easily identified in wasted or atrophic muscles of the limbs, particularly in the early stages of the disease [16,17].

### 2.2. Bulbar-Onset ALS

ALS with bulbar onset, or bulbar ALS, is the second leading phenotype with initial motor dysfunction in the bulbar region. Speech difficulties and frequent choking with concomitant hypersalivation are the cardinal presenting symptoms. Both LMN and UMN impairment are present, causing tongue wasting with fasciculations, facial spasticity and pseudobulbar affect in the early stages of the disease. Propagation to other spinal regions is evident later in the disease’s course [18]. Bulbar UMN involvement, also known as pseudobulbar palsy, can become clinically indicated by emotional lability, accompanied by excessive crying or laughing response to minor stimuli. This is termed the “pseudobulbar affect”. Other bulbar UMN signs include facial spasticity and slowed spastic movement of the tongue, whereas slurred speech, wasting of the tongue and fasciculations indicate bulbar LMN impairment [1,3]. Frontal release signs, such as the palmomental reflex, may also indicate bulbar involvement [19]. In contrast to the often used phrase “progressive bulbar palsy”, “ALS with bulbar onset” appears to be the more convenient term, as it represents a phenotypic description rather than a diagnostic label, suggesting no progression to other body regions [11].

### 2.3. Progressive Muscular Atrophy

Progressive muscular atrophy (PMA) presents with clinically isolated LMN impairment of the anterior horn cells and brainstem motor nuclei. However, subclinical UMN impairment can be detected in the early stages of the disease [20]. Although PMA tends to have a slower disease progression than classical ALS [21], 20 to 30% of the patients may develop ALS with clinically evident UMN impairment within 5 to 10 years from the disease’s onset. It is still controversially debated whether PMA should be considered a variant of ALS [20].

### 2.4. Primary Lateral Sclerosis

Primary lateral sclerosis (PLS) is characterized by progressive isolated UMN dysfunction detectable in at least two regions (thoracic region will not be considered) for at least two years. LMN dysfunction is absent, whereas minimal signs of denervation (positive sharp waves or fibrillation potentials) on electromyography (EMG) are permitted. Whether PLS constitutes a separate disease entity or rather represents a clinically benign variant of ALS remains controversial [22].

### 2.5. Flail-Arm-Syndrome

In flail-arm-syndrome (FAS), also known as Vulpian Bernhardt’s type, progressive, proximal and symmetrical weakness of both upper limbs caused by LMN impairment is predominantly apparent. Motor symptoms in bulbar muscles or lower limbs are unaffected from 12 to 20 months after the onset of upper limb symptoms [23,24]. LMN involvement is predominant, whereas UMN involvement can be occasionally present in lower limbs [25]. FAS represents a rather benign phenotype with a median survival time of 4 years and a 10 year survival rate of 17% [23].

### 2.6. Flail-Leg-Syndrome

Analogous to FAS, flail-leg-syndrome (FLS) presents itself with progressive and symmetrical weakness of both lower limbs, whereas distal muscle groups are typically affected and LMN involvement outweighs UMN involvement [24]. Other segments are clinically spared for a mean of 16 months after the disease’s onset. Unlike FAS, FLS has a similar prognosis as spinal-onset ALS, with a median survival time of 3 years and a 10-year survival rate of 13% [23].

### 2.7. Axial or Respiratory-Onset ALS

Axial-onset ALS initially presents itself with weakness of trunk muscles. Typically, paravertebral muscles are affected, resulting in bent posture, axial instability and dropped head syndrome [26]. However, weakness of the thoracic muscles can be rather difficult to recognize [8].

In respiratory-onset ALS patients suffer from dyspnoea and orthopnoea at the beginning of the disease, caused by weakness of the respiratory muscles and the diaphragm, which is also anatomically related to the thoracic region [27]. The prognosis is poor due to early respiratory failure and complications such as pneumonia [23,26,28]. 

There is no reliable method to detect thoracic UMN involvement [29]. However, brisk and deep abdominal reflexes, particularly with diminished or absent superficial abdominal reflexes, might be suggestive signs [30].

### 2.8. Hemiplegic ALS (Mill’s Syndrome)

This very rare phenotype is defined by slowly progressive, unilateral muscle weakness in the limbs alongside clinically predominant UMN signs, such as pathological deep tendon reflexes (DTR) and pyramidal tract signs. The onset may either occur in the upper limbs with subsequent descending propagation to the lower limbs or vice versa [31]. As is the case with PLS, there is an ongoing debate regarding whether Mill’s syndrome should be considered a distinct clinical entity in the spectrum of motor neuron diseases or an ALS variant [31,32,33]. 

## 3. Diagnostic Criteria

### 3.1. The El Escorial Criteria (1994) and Revised El Escorial Criteria (2000)

The El Escorial criteria were proposed as the first consensus diagnostic criteria for ALS in 1994 and were primarily designed for clinical trials and scientific research purposes [10]. However, they were increasingly applied in clinical practice due to the lack of any more reliable diagnostic procedures [34].

According to the criteria, clinical evidence of UMN and LMN impairment in four anatomically segmented body regions (bulbar, cervical, thoracic, lumbar) is surveyed for the diagnosis. Additionally, the progressive spreading of symptoms and the absence of electrophysiological and neuroimaging evidence of other causing diseases must be fulfilled. Patients are stratified into four categories of diagnostic certainty based on the number of affected regions and the extent of motor neuron involvement: suspected, possible, probable and definite ALS [10].

The criteria were revised (termed as revised El Escorial/Airlie House criteria) in 2000 to improve diagnostic sensitivity (Table 1). Diagnostic categories were refined with the following terms: clinically definite, clinically probable, clinically probable-laboratory supported and clinically possible ALS, while the category of suspected ALS was excluded. The electrophysiological examination was implemented to support clinical findings and identify LMN involvement, with signs of active and chronic denervation by EMG and the exclusion of motor neuropathy by nerve conduction studies [35].

### 3.2. The Awaji Criteria (2008)

The Awaji criteria, published in 2008, elaborated on the electrophysiological findings in the diagnosis of ALS (Table 1). Adapting the basic principles of the revised El Escorial criteria, evidence for chronic neurogenic damage in needle EMG is of equal significance as clinical signs for LMN involvement. Also, fasciculation potentials are equivalent to fibrillation potentials and positive sharp waves, implying that acute denervation if chronic neurogenic change on needle EMG is present. Consequently, the diagnostic categories were redetermined as follows: clinical definite ALS, clinically probable ALS and clinically possible ALS. The category of probable laboratory-supported ALS was discarded [36]. A meta-analysis proved a better diagnostic performance of the Awaji criteria with a sensitivity of 81.1% compared to the 62.2% of the revised El Escorial criteria, facilitating earlier diagnosis of ALS [37].

### 3.3. The Gold Coast Criteria (2020)

Although achieving improvement in diagnostic accuracy, both the revised El Escorial and the Awaji criteria are associated with various difficult aspects. Their complexity can be rather misleading and vulnerable to erroneous in their application, confirmed by a low test-retest as well as inter-rater reliability [38]. Also, the division into diagnostic categories may falsely suggest a predictive value about the actual occurrence of the disease. Furthermore, both criteria are limited in providing a prognostic value, as their diagnostic categories do not provide any relation to disease progression [39]. Ultimately, the category of possible ALS with isolated UMN signs in two regions exacerbates the debate about PLS being a separate entity or a prolonged form of ALS. Considering these issues, the Gold Coast criteria were introduced to further simplify the diagnostic approach by dichotomizing diagnostic categories into ALS and non-ALS (Table 2) [40]. Studies evaluating the feasibility of the Gold Coast criteria to date have shown an increase in diagnostic sensitivity with largely preserved high specificity [41].

## 4. Clinical Assessments of Disease Severity and Progression

To evaluate the functional status of patients, the ALS functional rating scale (ALSFRS) and its revised form (ALSFRS-R) were established. This self-assessment questionnaire contains items on bulbar functions, fine motor tasks, gross motor tasks and respiratory functions rated on a five-point scale from 0 to 4, with a maximum of 48 points [42,43]. The ALSFRS-R has become the gold standard tool to assess physical decline and rate of disease progression not only in clinical routine, but also in therapeutic trials. New staging systems, such as King’s staging system [44], Milano Torino Staging System (MiToS) [45] or Rasch overall ALS disability scale (ROADS) [46], have been recently developed to overcome methodological issues of the ALSFRS-R and improve reliability and measurement of therapeutic monitoring [47].

## 5. Pre-Symptomatic ALS

Since most recent pharmacological studies have yet failed to demonstrate an improvement of motor function or extend survival, much effort has been made to identify ALS patients in the early stages of the disease. The concept of pre-symptomatic ALS with mild motor impairment (MMI) has been discussed. Benatar and colleagues proposed a concept of ALS as a biological entity with a continuum of symptoms rather than a clinical syndrome alone. Therefore, there may be a pre-symptomatic phase with mild and/or unspecific symptoms that do not allow for a distinct differential diagnosis of MND until definite clinical signs of ALS occur and the ALS diagnosis according to recent diagnostic criteria is established [48]. In a small cohort of 20 pre-symptomatic patients with a known ALS gene mutation, there was found different mild motor symptoms, i.e., very mild focal weakness not resulting in disability, deep tendon hyperreflexia or EMG abnormalities, such as ongoing denervation. According to the underlying gene mutation, MMI onset takes place 1 month up to 10 years before ALS diagnosis has been established. While for some mutations the duration of the prodromal phase and disease was consistent (e.g., *SOD1 A4V* and *I113T*), a rather long prodrome over 4 years followed by a rapid disease course within about 15 months of the disease’s onset to permanent ventilation was reported. Conversely, *FUS* mutation carriers exhibit a relatively short prodromal stage, shorter than 1 year, followed by a disease course over 2.4 years [49]. Outside familial ALS, it remains a major concern to identify patients according to potential risk factors for developing sporadic ALS. Prospective population-based studies may be a useful tool to explore MMI in potential future ALS patients.

## 6. Cognitive and Behavioral Assessment

Historically defined as a pure motor neuron disease, ALS is now considered a complex multisystemic disorder not only occurring with motor impairment, but also with non-motor features, particularly cognitive and behavioral changes [50]. Cognitive and behavioral impairment have been extensively reported in ALS, occurring in up to 50% of patients, with approximately 10% exhibiting the full symptoms of FTD, predominantly the behavioral variant [51,52,53]. This is attributable to underlying aggregation of ubiquitinated transactive response DNA binding protein 43 (TDP-43), which has been identified as the common pathological hallmark and possible mechanism [54]. Foremost, deficits are observed in the domains of executive and language functions [51]. Since these cognitive and behavioral changes have an impact on prognosis, disease progression and caregiver burden, neuropsychological assessment has been emphasized in the diagnostic routine of ALS. As a result, the Strong criteria as diagnostic consensus criteria for the diagnosis of frontotemporal dysfunction in ALS were published. Patients meeting the criteria for cognitive or behavioral dysfunction are labelled as ALS with cognitive (ALS-ci) and behavioral impairment (ALS-bi) or both (ALS-bci) [55,56]. Criteria for FTD are implemented to stratify patients as ALS-FTD [57,58]. 

Many neuropsychological batteries, such as the Frontal Assessment Battery (FAB), the Montreal Cognitive Assessment Test (MoCa) or the Mini-mental State Assessment (MMSE) have been in clinical use. Disease-specific assessments have later been developed to counteract motor disabilities in ALS [59]. With the Edinburgh Cognitive and Behavioral ALS Screen (ECAS), a disease-specific assessment tool for determining the presence, severity and type of cognitive and behavioral changes in ALS, and its differentiation from other disorders is available. It consists of tasks testing for ALS-specific (executive function, language, fluency, social cognition) and non-ALS-specific (memory, visuospatial functions) cognitive changes and can be administered by neuropsychological and non-neuropsychological professionals within approximately 15 to 20 min [60]. A systematic review comparing different cognitive assessments for ALS entitled the ECAS promises to be the most suitable screen to detect cognitive or behavioral changes in ALS [59].

## 7. Technical Diagnostic Tools

Electrodiagnostic (ED) studies are established and fundamental for the correct diagnosis of MND, particularly for the exclusion of disease mimics such as multifocal motor neuropathy (MMN). Nerve conduction studies are required to demonstrate an affection of motor nerves, indicated by reduced compound muscle action potential, prolonged distal motor latency and decreased conduction velocity without evidence for conduction blocks. This is complemented by needle EMG examination evaluating signs of acute and chronic denervation. In recent years, much effort has been invested in the development of more sophisticated approaches to diagnose MND with higher accuracy at an early stage in the course of the disease. As to the point of differentiation from disease mimics, the nerve ultrasound has become a powerful tool to aid in the diagnostic work-up when ED studies remain inconclusive [61]. While MMN, for instance, usually presents itself with enlarged cross-sectional areas (CSA) of the nerves, this is not found in MND [62]. Indeed, the CSA appears reduced in MND, which is associated with clinical affection of the respective innervated muscle, yielding a higher diagnostic accuracy [63]. Analysis of the vagus nerve (VN) has become another interesting application of nerve ultrasound in ALS, showing a reduced CSA of the VN on both sides in ALS, independent of clinical symptoms [64,65]. This could add another pathophysiological hint as to the involvement of the autonomous system, which was also reported to be affected in ALS, although only to a mild extent and without a correlation with disease severity [6,65]. To further determine structural deficits of the peripheral nervous system suggestive of axonal degeneration in ALS, different magnetic resonance imaging (MRI) techniques were reported to add diagnostic value and work as a surrogate for disease progression, as measured by the ALSFRS [66,67]. Beyond the pure structural point of view, modern MRI sequences allow a functional–connectivity assessment, such as DTI (diffusion tensor imaging), which has been thoroughly investigated as a potential biomarker for ALS in the past years [67]. It is a measure for the unguided diffusion of water according to the Brownian movement, allowing assessment of the direction of the flux. It is usually numerically expressed by the fractional anisotropy, which can vary between 0 (pure spherical diffusion) and 1 (diffusion is strictly directed in one direction). More importantly, this technique has been extensively investigated in the field of MND and applied to determine the deterioration of both the peripheral and central motor nervous systems in ALS [65,66,67]. While routine MRI of the central nervous system is essential for regular diagnostic work-up, it often appears unremarkable, shows T2 hyperintensities along the corticospinal tract (CST) or shows atrophy of the precentral gyrus, which are, however, of unclear specificity [68]. In contrast, defined multiparametric MRI measurements, including DTI, allow for a much more accurate and reliable diagnostic workflow. However, it needs to be highly standardized [69]. On the other hand, these different imaging modalities offer a vast range of different data types that can be integrated into a multiparametric model acquired by machine learning algorithms, which could be a very powerful approach to making an unsupervised diagnosis based on neuroimaging data [70]. However, such elaborated MRI approaches require a high level of expertise and technical equipment, which is a limiting factor. Nonetheless, multiparametric MRI assessment including DTI should be accounted as a facultative biomarker for the diagnosis of MND in the future. 

Furthermore, in addition to the growing importance of fluid biomarkers for neuroinflammation in ALS, positron emission tomography (PET) imaging with highly specialized tracers indicating glial activation has caught attention. Standard [^18^F]Fluorodeoxyglucose-PET (FDG-PET) demonstrates distinct patterns of hypometabolism in the frontotemporal cortex [71,72] and diverse areas of hypermetabolism (e.g., spinal cord, cerebellum [72,73,74]). However, tracers indicating inflammatory processes might reflect the disease course in a much more reliable way. The [11C]-PBR28 tracer binds to the 18pkD translocator protein (TSPO), which is located in glial cells and can indicate inflammatory activation. TSPO is enriched in the precentral gyrus of patients with ALS, correlating with the disease burden of upper motor neuron symptoms [74]. Although having shown no correlation to disease progression in a longitudinal observation of patients with ALS, it still shows potential as a very specific biomarker. Broader studies including patients with fast disease progression and correlations to neuroinflammatory biomarkers are required to foster the concept of neuroinflammation as a diagnostic tool in ALS.

## 8. Genetic Testing

Most ALS cases are sporadic and cannot be accounted for by a single genetic mutation, but a multitude of monogenic causes for ALS have been identified [75]. They are thought to constitute 5 to 10% of all ALS cases. More than 30 potentially causative genes have been identified, and the large majority are inherited in an autosomal-dominant manner. Mutations in most of these genes are very infrequent and most Mendelian ALS cases are due to mutations in chromosome 9 open reading frame 72 (*C9orf72*), superoxide dismutase 1 (*SOD1*), fused-in sarcoma (*FUS*) and TAR DNA binding protein (*TARDBP*). It is important to note that up to 20% of patients with a negative family history have been reported to harbor a causative genetic variant [76,77]. In a survey published in 2017, 90% of caregivers reported offering genetic testing to patients with familial ALS and 50% to patients with sporadic ALS. Most commonly, *SOD1*, *C9orf72*, *TARDBP*, and *FUS* were tested [78]. With more widespread availability of genetic testing in general and next-generation sequencing (NGS) in particular, it seems likely that the number of caregivers offering genetic testing has increased and that NGS approaches are used more frequently. The diagnostic yield is certainly higher using these approaches and progress in the analysis of sequencing data will lead to the identification of even more variants with effect on ALS risk, especially structural variations and intronic variants [79,80]. 

In contrast to other rare diseases, in the vast majority of ALS cases genetic testing will not be used to establish a diagnosis. However, genetic testing in patients with ALS does have a number of important implications:

1. Exclusion of genetic disorders mimicking ALS, such as spinal and bulbar muscular atrophy (SBMA) which is caused by a polyglutamine expansion in the androgen receptor (*AR*) gene and can be mistaken for LMN-predominant ALS [81]. Other examples include, but are not limited to, adult-onset spinal muscular atrophy, a number of hereditary spastic paraparesis (HSP) subtypes and adult polyglucosan body disease (APBD) [82];
2. Identification of patients who are eligible for trials targeting specific mutated genes, such as *SOD1*, *C9orf72*, Ataxin 2 (*ATXN2*) and *FUS* [83]. For patients with *SOD1* mutations, tofersen, an antisense oligonucleotide reducing SOD1 protein synthesis, was shown to reduce neurofilament light chain levels in plasma and is already available in many countries in an early access program [84,85];
3. Counselling of family members concerning predictive testing. Predictive testing always needs thorough counselling, especially when there are no measures to prevent the disease, as is currently the case for ALS [86,87]. However, family members may benefit from the knowledge gained from genetic testing, either because they are relieved when they do not harbor the mutation or because they are able to plan ahead for specific life decisions. This does also include preimplantation genetic diagnosis if a desire to have children is present [88]. Also, the exemplary ATLAS trial is already trying to closely follow up asymptomatic carriers of disease-causing mutations to initiate therapy on the basis of biomarker-defined phenoconversion and before the advent of clinical symptoms for an optimal disease-modifying effect [89]. Approaches like these will likely be applied more often as more gene-specific therapies become available;
4. Identification of genetic mutations may help in the prediction of the clinical course of the disease, which is of importance for patient and caregiver counselling (see prediction models section).

It should be mentioned that, with the advent of NGS techniques, the identification of variants of uncertain significance (VUS) can be challenging when counselling patients and relatives [90]. In one study, 47% of variants in ALS patients with potential functional significance were classified as VUS [91]. Segregation analysis, consultation of population databases and even functional analysis will often leave uncertainty concerning the pathogenicity of VUS and thorough pre-test counselling is therefore of utmost importance so patients can anticipate possible results [92].

Apart from disease-causing mutations, genome-wide association studies (GWAS) have identified a number of variants that modify the risk to develop ALS. They offer valuable insights into ALS pathogenesis and may help in the identification of treatment targets, but are not currently of additional diagnostic value [93]. In the future, polygenic risk scores (PRS) may also be of help in predicting disease progression, as one study already hinted at an association of a PRS and cognitive decline in ALS, or help stratify ALS patients according to underlying disease processes using pathway-specific PRS, as has been suggested for Parkinson’s disease [94,95].

## 9. Diagnostic and Prognostic Value of Fluid Biomarkers

### 9.1. Neurofilaments

Neurofilaments (Nf) emerged as one of the most promising biomarkers of ALS in the recent past. Considered an indicator of ongoing neuronal or axonal injury, the role of Nf in the pathophysiology of ALS remains unclear [96]. Elevated Nf can be found in several neurological diseases, including neurodegenerative disorders such as atypical Parkinson syndromes or FTD, as well as infectious or inflammatory disorders, such as multiple sclerosis (MS) or Creutzfeld-Jakob disease (CJD) [97,98]. Therefore, interpretation of Nf elevation should always be performed in the context of the primary suspected diagnosis and clinical finding. As of now, examination of the neurofilament light chain (NfL) and the phosphorylated heavy chain of the neurofilament (pNfH), both in the cerebrospinal fluid (CSF) and serum, are established methods and comparable with regard to diagnostic utility. Both NfL and pNfH values in the CSF and serum strongly correlate [99,100,101]. Numerous studies found a significant elevation of Nf in ALS patients compared to both healthy and disease controls [100,101,102,103,104,105]. Additionally, the site of symptom onset or presence of ALS-related gene mutations, such as *C9Orf72*, *SOD1* or *FUS*, are associated with higher Nf values. ALS patients with known bulbar onset or with a mutation in the *C9Orf72* tend to have higher Nf levels [101,106,107]. The same applies to ALS patients with more regions involved or present upper motor neuron signs at the time of diagnosis [100]. 

One major issue in the diagnosis of ALS is the date of diagnosis, since a proper diagnosis of ALS is established up to 10 to 12 months after the first symptoms emerge [108]. In pre-symptomatic patients with a known ALS-related mutation, elevated Nf values can be found up to 12 months before patients develop the first ALS-related symptoms [99,109]. Before that, Nf values are comparable to healthy controls [99]. After symptom onset, the longitudinal analysis revealed almost stable values for neurofilaments over the course of the disease [99,101,109]. The increase of Nf from baseline prior to symptom onset depends on the respective mutation found, i.e., in ALS patients with known SOD1 mutation, approximately 6 to 12 months in advance of the first emergence of ALS symptoms. This is compared to 2 to 3.5 years in ALS patients with mutations in the *FUS* gene or *C9Orf72* with hexanucleotide (G_4_C_2_)*_n_* repeat expansion (HRE) [109]. Although these measurements are currently only available for a few patients with inherited ALS, they can most likely apply to sporadic ALS patients to an extent, as neuronal and axonal injury precedes the onset of motor symptoms in sporadic patients as well. Nf may shorten the diagnostic delay by up to 3 months in patients with suspected ALS [110]. 

The diagnostic sensitivity and specificity of Nf measurement depend, among other things, on the measurement of either NfL or pNfH and the assay used, yet efforts have been made to standardize procedures for comparable results among different laboratories [103]. Steinacker and colleagues first demonstrated a sensitivity of 77% and a specificity of 88% for CSF NfL values using a cut-off of 2200 pg/mL to distinguish ALS patients from ALS mimics. For CSF pNfH values above 560 pg/mL, a robust sensitivity of 83% and a specificity of 80% were found. Subsequent studies confirmed the usability of NfL and pNfH by applying comparable cut-off values for CSF NfL and CSF pNfH in the differential diagnosis of ALS (Table 3). The examination of serum NfL demonstrated comparable results for sensitivity and specificity [111]. However, pNfH examination in the serum seems to be inferior regarding differential diagnosis [112]. Besides the differential diagnostic value of the Nf measurement, several studies addressed the prognostic and therapeutic aspects of Nf in ALS patients. Elevated Nf positively correlates with disease progression rate [100,104]. Conversely, ALS patients with longer disease duration display lower Nf levels [103]. Additionally, high Nf levels are associated with shorter survival [101,104]. However, findings considering the therapeutic implications of Nf are ambiguous. In both phase 1/2 and phase 3 of tofersen trials, an antisense oligonucleotide (ASO) targeting *SOD1* messenger RNA (mRNA) transcripts in ALS associated with mutations in *SOD1*, there was a significant reduction of NfL levels in the CSF and plasma, but no improvement in clinical endpoints could be demonstrated [84,85]. On the other hand, Riluzole, currently the only approved treatment for ALS, does not alter Nf levels after treatment initiation [113]. Nevertheless, findings regarding Nf in the therapy of other neurologic disorders are encouraging. In relapse-remitting MS, Nf may serve as a marker for treatment response for different disease-modifying therapies [114]. In other motor neuron diseases, such as spinal muscular atrophy (SMA), a significant reduction of plasma pNfH was found after treatment initiation with nusinersen, an ASO targeting *SMN1* splicing, in children with SMA [115]. Therefore, the role of Nf as a marker for therapeutic response remains to be determined.

### 9.2. Inflammatory Biomarkers 

The pathophysiology of ALS is characterized not only by neurodegenerative but also by inflammatory processes involving glial cells of the central nervous system and peripheral circulating immune cells [116]. Genetic alterations linked to ALS, e.g., in *SOD1* and *C9orf72*, are also associated with the dysregulation of immune processes [117,118]. However, the dysfunction of autophagy and glial cells was also present in ALS patients without these genetic alterations [119]. It is assumed that anti-inflammatory processes predominate at the onset of the disease, while proinflammatory processes become relevant in later stages, accelerating motor neuron injury [120,121]. This marks the relevance of inflammatory biomarkers, which promise to provide information on disease stage, progression rate as well as pathophysiological and potential protective mechanisms.

Several studies have investigated patterns of blood immune cells, such as granulocytes and T cells, as possible diagnostic biomarkers in ALS patients, finding altered leukocyte phenotypes [122,123]. Others found differentially regulated soluble factors such as interleukin (IL)-6, IL-8, tumor necrosis factor (TNF) and interferons [124,125,126]. They generally suggest differential immune regulation in ALS compared to healthy individuals. However, findings are heterogeneous and quite variable between studies and different methodological approaches. It is especially difficult to distinguish ALS from mimics, as they are often inflammatory diseases. More detailed data were provided by studies on a transcriptional level, including the finding that proinflammatory gene profiles had higher expression levels of IL-8, FBJ murine osteosarcoma viral oncogene homolog B (*FOSB*), cluster of differentiation 83 (*CD83*), suppressor of cytokine signaling 3 (*SOCS3*), chemokine (C-X-C motif) ligand 1 (*CXCL1*) and *CXCL2* in monocytes of ALS patients compared to healthy controls [127]. Nevertheless, these inflammatory diagnostic biomarkers are far from making their way into clinical use. Similarly, they have not been proven to provide a prognostic value. Most studies failed to find a significant correlation between common clinical progression parameters such as the ALSFRS-R and blood concentrations of pro- or anti-inflammatory cytokines in ALS patients [124,128]. Other groups found correlations between the ALSFRS-R and survival on the one hand and seemingly protective monocyte and T-cell immune profiles on the other [122]. In addition to this, a higher number of pro-inflammatory differentially-expressed genes in monocytes of ALS patients was associated with faster disease progression [127]. Another potential inflammatory biomarker for ALS is neopterin, which is secreted by macrophages under interferon-gamma influence from stimulated T lymphocytes, is an indicator of general immune system activation and is renally excreted. In urine, it can thus be examined non-invasively without great effort. Higher concentrations were found in ALS patients than in patients with other neurological diseases, such as multiple sclerosis, and in healthy controls [129]. A higher neopterin level in ALS patients was associated with more severe symptoms evaluated by the ALSFRS-R [130].

In addition to peripherally circulating inflammatory biomarkers, there are CNS-specific ones that represent microglial and astrocyte-derived inflammation, which, however, are more difficult to access and measure. Up-regulation of activated microglia and astrocytes producing pro-inflammatory cytokines was found in the spinal cord tissue of ALS patients [119]. *SOD1*-mutated mouse microglia were found to express predominantly anti-inflammatory markers like chitinase-like 3 (*Ym1*), cluster of differentiation 163 (*CD163*) and brain-derived neurotrophic factor (*BDNF*) mRNA and fewer proinflammatory markers like NADPH oxidase 2 (*Nox2*) mRNA at disease onset than later in disease progression, consistent with observations of other peripheral inflammatory markers during disease course [131]. Astrocytes and microglia have been shown to interact and alter each other’s phenotype through the release of inflammatory mediators affecting disease progression in a mouse model [132]. As a representation of neuroinflammatory involvement not only in the spinal cord, but also in the motor cortex in early stages of ALS with TDP-43 pathology, activated astrocytes and microglia were detected in this brain area in patients and a TDP-43 mouse model. It was also demonstrated that cells of the primary motor cortex express the monocyte chemoattractant protein-1 (MCP1), a ligand for C-C chemokine receptor 2+ (CCR2+) monocytes infiltrating the CNS, driving the immune response in this area [133]. Recently, some approaches detected the above-explained inflammatory processes non-invasively using functional imaging, e.g., using positron emission tomography [134].

### 9.3. Chitinase, Tau Protein, TDP-43, Creatine Kinase and Other Fluid Biomarkers

Elevations of chitinases (CHIs) and chitinase-like proteins (CLPs) have also been found in the CSF of ALS patients. CHIs are hydrolytic enzymes, widely distributed in nature, that metabolize chitin, the most abundant polysaccharide in nature and essential structural component of several organisms, including arthropods, protozoan parasites, nematodes, bacteria and fungi. Despite the absence of endogenous chitin, mammals express true CHIs with enzymatic activity and homologous structurally related CLPs lacking enzymatic activity but bind chitin with high affinity [135]. Despite its implication in several neurological diseases, the function of CHIs and CLPs in the CNS is still not completely understood. Chitotriosidase-1 (CHIT1) has been found only in microglia and CNS infiltrating peripheral macrophages [136]. Chitinase-3-like protein 1 (CHI3L1) has been mostly found in reactive astrocytes [137]. Little is known about the role of Chitinase-3-like protein 2 (CHI3L2) in physiologic and pathologic conditions. Increased CHIT1, CHI3L2 and CHI3L2 expression or CSF levels have been reported in various neuroinflammatory conditions [138]. CHIs and CLPs have been recently investigated. Thompson and colleagues showed that CHIT1, CHI3L1 and CHI3L2 were elevated in the CSF of patients with ALS compared with healthy controls and ALS-mimics. CHIT1 and CHI3L2 were elevated in ALS compared with PLS [139,140]. Additionally, the CHIT1 response appears to be an attribute of the late pre-symptomatic to early symptomatic phases in patients carrying mutations in *C9orf72* or *SOD1* [141]. 

Other markers commonly used for the diagnosis of other neurodegenerative diseases have also been studied in the context of ALS. For example, the microtubule-associated protein Tau, commonly found elevated in Alzheimer’s disease, increases significantly in the CSF of ALS patients. Significantly higher levels of total Tau (tTau) and lower phosphorylated Tau (pTau)/tTau ratio have been found in ALS patients in comparison with healthy controls in observational studies [142]. β-Amyloid, another Alzheimer’s disease-related biomarker, is elevated in the CSF of ALS patients and seems to predict shorter survivals [143] correlating with the ALSFRS-R at baseline [144]. On the other hand, the soluble amyloid precursor protein (sAPPβ) appears reduced in the CSF of ALS and FTD patients, correlating with cognitive performance [145]. TDP-43 has also demonstrated some prognostic value in ALS patients. Several studies have reported elevated CSF TDP-43 levels in patients with ALS [146]. Similarly, significantly increased levels of TDP-43 and pTDP-43 have been found in plasma of the ALS patients. Especially, the pTDP-43/TDP-43 ratio appears to distinguish individuals with ALS from healthy controls [147]. High levels of miR-181, a highly conserved non-coding RNA molecule enriched in neurons, predict a greater than a two-fold risk of death in ALS patients. The molecule miR-181 performed similarly to NfL, and when combined, miR-181 + NfL show a superior prognostic value [148].

Concerning non-neuronal related biomarkers, higher levels of creatine kinase (CK) are often found in ALS patients, especially in those with slow progression, correlating to lower ALSFRS-R scores. Higher CK blood concentration is likely linked to longer survival [149]. In contrast to the cardiac troponin I (cTnI), serum concentration of cardiac troponin T (cTnT) is elevated in the serum of ALS patients. Both are common biomarkers in the initial approach of myocardial infarction. This is especially true in patients with a spinal onset (AUC 0.87; 0.78–0.94), and it can thus differentiate ALS from other neurodegenerative diseases and ALS mimics [150]. CSF levels of the basic fibroblast growth factor (bFGF) are increased in ALS and correlate with disease duration and survival [151]. Similarly, the perivascular fibroblast marker Secreted Phosphoprotein 1 (SSP1, Osteopontin) increased in plasma of ALS patients in four independent cohorts. Increased levels of SPP1 at disease diagnosis predicted shorter survival as well [152].

### 9.4. Frontiers in Fluid Biomarkers

Despite the value as a diagnostic tool showed by increasing research on Nf, many other neurological disorders present elevated Nf in serum and CSF, decreasing its specificity in the ALS diagnosis. Currently, great effort is exerted in clinical research to find ALS-specific biomarkers that indicate the onset of pathological events in pre-symptomatic or prodromal phases of the disease. Among the most promising is the translation products of the *C9orf72* intronic expansion, poly-GP dipeptide repeats, which are increased in the CSF of pre-symptomatic patients of *C9orf72*-associated ALS. Similarly, higher levels of the poly-GP proteins were also found in peripheral mononuclear cells of pre-symptomatic *C9orf72* mutation carriers [153,154]. Recently, it was found that the nuclear TDP-43 suppresses cryptic exon-splicing events of some ALS-associated genes, such as *UNC13A*. This repression is lost in the ALS/FTD pathology, as extranuclear TDP-43 is a specific hallmark of these disorders. The early identification of such cryptic exon-splicing variants or their translational products represents one of the most promising and specific biomarkers for identifying disease onset [155]. 

## 10. Predictive Models

“Prognosis can no longer be relegated behind diagnosis and therapy in high-quality neurologic care” [156]. With a diagnosis of ALS, the question of prognosis almost automatically arises. However, the disease course is highly variable. This poses a problem when discussing prognosis with the individual patient, but also when dealing with high variability in the design and evaluation of disease-modifying trials [157]. Biomarkers such as neurofilaments and rating scales such as the ALSFRS-R at baseline, the ALSFRS-R decline from disease onset to the test date, the initial clinical presentation or specific genetic mutations are all individually associated with disease progression and survival time [158,159,160,161,162]. The multitude of parameters that can be evaluated in patients with ALS calls for approaches that incorporate many factors influencing the disease course to provide a more accurate estimate of how the disease will likely progress in the individual patient. The ENCALS survival prediction model uses eight predictors to define five groups with distinct survival outcome, which was defined as the time between symptom onset and non-invasive ventilation > 23 h/day, tracheostomy or death [157]. 

Clinical parameters, such as bulbar vs. non-bulbar onset, forced vital capacity, the age of onset and the diagnostic delay and the presence of *C9orf72* repeat expansion, were used in the model. Patients with a predicted brief disease course had a median of 17.7 months from symptom onset to the composite survival outcome, while the median was 91.0 months for the group with a very long course.

A qualitative study looking at the impact of personalized prognosis using the ENCALS survival prediction model found that it can be discussed with “minimal adverse emotional impact” and may help facilitate planning of the future [163]. However, the quotes of patients, relatives and caregivers in reaction to the prediction, probably not unexpectedly, show varying reactions. More research is needed to assess the impact of personal prediction models in ALS.

Additionally, while the ENCALS model at least incorporated one non-clinical parameter by using the *C9orf72* repeat expansion as a predictor, many studies trying to predict ALS or trying to identify ALS subgroups are still only or mostly using clinical parameters [164,165]. We are convinced that, in the future, the combination of clinical signs, genetic testing and biomarkers, such as neurofilaments, will offer the patient an informed prognostic estimation after establishing a diagnosis of ALS.

Establishing and refining experimental disease models will be crucial for obtaining information about the underlying heterogenous disease-causing mechanisms and identifying new potential diagnostic and therapeutic targets in ALS, particularly in its sporadic forms. While transgenic animal models for familiar ALS with the disease-causing mutation have provided insights into pathogenesis and potential therapeutic targets, identifying the underlying disease and causes of sporadic forms remains challenging. Currently, various in vitro cellular models with induced pluripotent stem cells (iPSCs) [166] and organoids [167] from donors with sporadic ALS show phenotypic differences in the pattern of neuronal organization and degeneration, protein aggregation, cell death, as well as in onset and progression [168]. The further development of these methods in combination with advanced omics technologies, such as single-cell sequencing and Deep Learning algorithms, will allow precise, accurate and reliable decoding of patient-specific cellular and genetic dysfunction, leading not only to an individual molecular diagnosis but a solid predictive model for applying personalized therapies [169,170].

## 11. Conclusions

**Figure 2:**
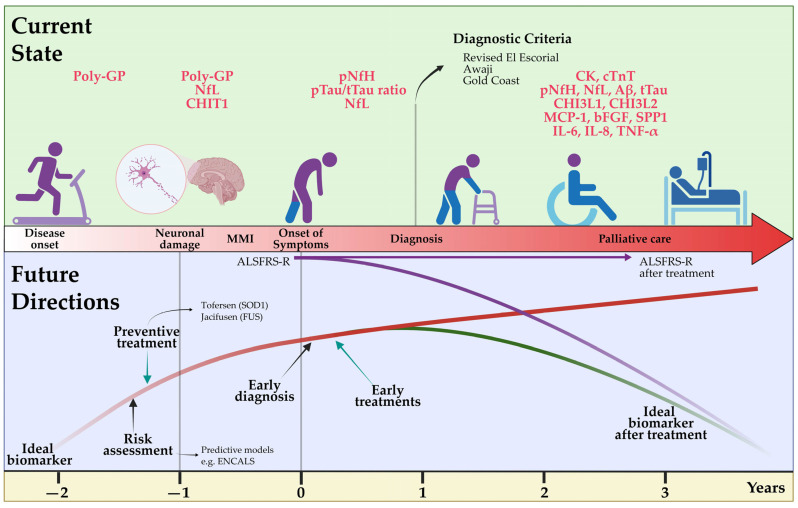
Current state of and future directions for the diagnosis of amyotrophic lateral sclerosis. A delayed diagnosis of ALS is still currently performed using clinical criteria such as Revised El Escorial, but the determination of fluid biomarkers such as Nf is accelerating an earlier identification of the disease. In the future, the availability of more specific biomarkers related to disease pathology onset will allow early risk assessment and definitive diagnosis in patients in prodromal phases. Additionally, an ideal biomarker should develop preventive therapies and monitor disease activity in patients under novel causative therapies. ALSFRS-R: Revised Amyotrophic Lateral Sclerosis Functional Rating Scale; Aβ: Amyloid β; bFGF: basic fibroblast growth factor; CHI3L1: chitinase-3-like protein 1; CHI3L2: chitinase-3-like protein 2; CHIT1: chitotriosidase 1; CK: creatinine kinase; cTnT: cardiac troponin T; IL-6: interleukin-6; IL-8: interleukin-18; MCP-1: monocyte chemoattractant protein-1; MMI: mild motor impairment; NfL: neurofilament light chain; pNfH: phosphorylated neurofilament heavy chain; Poly-GP: arginine containing dipeptide repeat polymers; pTau: phosphorylated tau; SPP1: secreted phosphoprotein 1; TNF-α: tumor necrosis factor; t-Tau: total tau. Created by Castro-Gomez with “BioRender.com”; accessed on 04 February 2023.

The diagnosis of ALS is currently primarily based on clinical aspects as options for early detection, such as biomarkers with high diagnostic accuracy, are lacking. The ongoing development of new and innovative diagnostic tools, however, promises major advances that will fundamentally impact future clinical practice and research in this field (Figure 2). Expert clinical assessment remains essential in diagnosing ALS. Its heterogeneous clinical presentation and multisystemic complexity can be challenging and requires interdisciplinary assessment of cognitive and behavioral aspects in addition to pure motor impairments. Electrophysiological assessments as well as imaging techniques, such as functional MRI and PET, are becoming increasingly sensitive and can aid in early diagnosis and, in particular, differentiation from related diseases. In addition to established Nf in the diagnostic workup, other biomarkers of several systems, ranging from inflammatory to degenerative types, are emerging. Ideally, biomarkers need to be easy to obtain and minimally invasive, improve diagnostic accuracy, facilitate early detection of ALS and enable the monitoring of disease progression. This would aid in enrolling patients in new clinical trials even in pre-symptomatic phases, evaluating new treatments and optimizing treatment plans and disease management. Early identification of affected and pre-symptomatic individuals may prevent prolonged diagnostic procedures in the future and greatly improve the potential efficacy of therapeutics by extending and advancing the treatment window. Genetic testing is becoming more feasible and frequently used. It promises a better endophenotypic classification of patients based on their neurobiological disease correlates and thus may enable targeted treatment and improved prediction of individual disease courses. Ultimately, the integration of various diagnostic methods and all collected patient data will be key to deriving patterns towards more personalized diagnostics with the future opportunity of evaluating individual prognosis as well as directing patients towards treatment studies or approved therapeutic strategies. 

## Figures and Tables

**Table 1:** The revised El Escorial criteria (2000) and the Awaji criteria (2008).

**Clinically definite ALS**	Clinical or electrophysiological * evidence of UMN and LMN involvement in bulbar region and ≥2 spinal regions
	*or*
	Clinical or electrophysiological * evidence of UMN and LMN involvement in 3 spinal regions
**Clinically probable ALS**	Clinical or electrophysiological * evidence of UMN and LMN involvement in ≥2 regions with UMN signs rostral to LMN signs
**Clinically probable** **Laboratory-supported ALS ^⧫^**	Clinical evidence of UMN and LMN involvement in 1 region
	*or*
	Clinical evidence of isolated UMN involvement in 1 region with electrophysiological evidence of LMN involvement in ≥2 regions
**Clinically possible ALS**	Clinical or electrophysiological * evidence of UMN and LMN involvement in 1 region
	*or*
	Evidence of isolated UMN involvement ≥2 regions
	*or*
	Evidence of LMN involvement rostral to UMN involvement

ALS: Amyotrophic lateral sclerosis; UMN: upper motor neuron; LMN: lower motor neuron; * additional feature for Awaji criteria; ^⧫^ only included in revised El Escorial criteria.

**Table 2:** The Gold Coast criteria (2020).

Progressive motor impairment
*and*
Clinical or electrophysiological UMN and LMN involvement in ≥1 region *or* only LMN involvement in ≥2 regions
*and*
Exclusion of other diseases

UMN: upper motor neuron; LMN: lower motor neuron.

**Table 3:** Sensitivity and specificity of Neurofilament in CSF. Reference values for neurofilaments in the cerebrospinal fluid. Demonstrated are sensitivity, specificity and positive and negative predictive values for different neurofilament cut-off values for distinguishing ALS patients from either ALS mimics, disease controls or healthy controls.

Neurofilament	Cut-Off [pg/mL]	Sensitivity (95% CI) [%]	Specificity (95% CI) [%]	NPV (95% CI) [%]	PPV (95% CI) [%]	ALS vs.	Study
NfL	>2200	77 (71–82)	88 (79–94)	56 (48–65)	95 (91–98)	ALS mimics	[105]
	>2200	-	85 (79–90)	75 (69–80)	87 (81–91)	Other controls	[105]
pNfH	>560	83 (78–88)	80 (70–88)	62 (52–71)	93 (88–95)	ALS mimics	[105]
	>560	-	77 (71–83)	79 (72–84)	82 (77–86)	Other controls	[105]
NfL	>3819	88.4 (78.8–94)	84.7 (76.8–90.2)	-	-	Disease controls	[100]
	>2453	85.4	78	-	-	ALS mimics	[100]
pNfH	>618	94.2 (86–97.7)	74.8 (66–81.9)	-	-	Disease controls	[100]
	>768	90.7	88	76	-	ALS mimics	[100]
NfL	1431	79 (66.1–87.6)	86.4 (75.7–93.6	-	-	Controls	[103]
pNFH	568.5	78.7 (67.7–87.3)	93.3 (85.1–97.8)	-	-	Controls	[103]

## Data Availability

No new experimental data were created or analyzed in this study. Data sharing is not applicable to this article.

## Abbreviations

ALSFRS-R	Revised Amyotrophic Lateral Sclerosis Functional Rating Scale
ADM	abductor digiti minimi
ALS	amyotrophic lateral sclerosis
ALS-bci	ALS with cognitive and behavioral impairment
ALS-bi	ALS with behavioral impairment
ALS-ci	ALS with cognitive impariment
APB	abductor pollicis brevis
APBD	adult polyglucosan body disease
AR	androgen receptor
ASO	antisense oligonucleotide
ATXN2	Ataxin 2
Aβ	Amyloid β
bFGF	basic fibroblast growth factor
C9orf72	chromosome 9 open reading frame 72
CD83	cluster of differentiation 83
CHI3L1	chitinase-3-like protein 1
CHI3L2	chitinase-3-like protein 2
CHIT1	chitotriosidase 1
CJD	Creutzfeld-Jakob disease
CK	creatine kinase
CLP	chitinase-like proteins
CSA	cross-sectional areas
CSF	cerebrospinal fluid
CST	corticospinal tract
cTnT	cardiac troponin T
CXCL1	chemokine (C-X-C motif) ligand 1
CXCL2	chemokine (C-X-C motif) ligand 2
DTI	diffusion tensor imaging
DTR	deep tendon reflex
ECAS	Edinburgh Cognitive and Behavioral ALS Screen
ED	electrodiagnostic
EMG	electromyography
FAB	Frontal Assessment Battery
FAS	flail-arm-syndrome
FDG-PET	[^18^F]Fluorodeoxyglucose-PET
FDI	first dorsal interosseus
FLS	flail-leg-syndrome
FOSB	FBJ murine osteosarcoma viral oncogene homolog B
FTD	Frontotemporal dementia
FUS	fused-in sarcoma
GWAS	Genome-wide association studies
HRE	hexanucleotide (G_4_C_2_)*_n_* repeat expansion
HSP	hereditary spastic paraparesis
IL-18	interleukin-18
IL-8	interleukin-8
iPSCs	induced pluripotent stem cells
LMN	lower motor neurons
MCP-1	monocyte chemoattractant protein-1
MiToS	Milano Torino Staging System
MMI	mild motor impairment
MMN	multifocal motor neuropathy
MMSE	Mini Mental State Assessment
MND	motor neuron disease
MoCa	Montreal Cognitive Assessment Test
MRI	magnetic resonance imaging
MS	multiple sclerosis
Nf	neurofilaments
NfL	neurofilament light chain
NGS	next-generation sequencing
PET	positron emission tomography
PLS	primary lateral sclerosis
PMA	progressive muscular atrophy
pNfH	phosphorylated neurofilament heavy chain
Poly-GP	arginine containing dipeptide repeat polymers
PRS	polygenic risk scores
pTau	phosphorylated Tau
ROADS	Rasch overall ALS disability scale
sAPPβ	soluble amyloid precursor protein
SBMA	spinal and bulbar muscular atrophy
SMA	spinal muscular atrophy
SOCS3	suppressor of cytokine signaling 3
SOD1	superoxide dismutase 1
SPP1	secreted Phosphoprotein 1
TARDBP	TAR DNA binding protein
TDP-43	transactive response DNA binding protein 43
TNF-α	tumor necrosis factor
TSPO	18pkD translocator protein
t-Tau	total Tau
UMN	upper motor neurons
Vn	vagus nerve
